# Postoperative pain of impacted mandibular third molar surgery performed by general dental practitioners – a multicenter study

**DOI:** 10.4317/jced.62870

**Published:** 2025-08-01

**Authors:** Fernando J. Mota de Almeida, Yvette Amba Kindlund, Robert Lundqvist, Angelika Lantto

**Affiliations:** 1 PhD, Tandvårdens Kompetenscentrum, Norrbotten Public Dental Service, Region Norrbotten, Luleå, Sweden; 2 DDS, Private practice, Luleå, Sweden; 3 Techn lic, Department of Public Health and Clinical Medicine, Sunderby Research Unit - Norrbotten, Umeå University, Sweden; 4 PhD, Tandvårdens Kompetenscentrum, Norrbotten Public Dental Service, Region Norrbotten, Luleå, Sweden

## Abstract

**Background:**

Impacted third molars are common and often require surgical removal, which can lead to postoperative complications, particularly pain. While pain has been extensively studied in specialist settings, data from general dental practice remains limited. This study evaluated postoperative pain over seven days following mandibular third molar surgery performed by general dental practitioners (GDPs), who conduct a significant number of these procedures.

**Material and Methods:**

A prospective multi-center cohort study was conducted in three public dental clinics in Luleå, Sweden, from October 2022 to December 2023. Participants (≥18 years old) undergoing mandibular third molar surgery were included, while those requiring referral to an oral maxillofacial surgeon were excluded. Pain intensity was self-reported daily using a numerical rating scale (NRS) from 0–10. Statistical analyses included descriptive statistics, chi-square tests, and t-tests.

**Results:**

Of 133 enrolled participants, 111 submitted valid pain assessments. Pain peaked on the day of surgery (Day 0), with 4% reporting the worst imaginable pain (NRS 10), 34% severe pain (NRS 7–9), and 35% moderate pain (NRS 4–6). Pain significantly declined by Day 1 (*p*<0.001) and continued to decrease throughout the week. Female participants reported higher pain scores (statistically significant on Days 2–4) and used more analgesics. Sedated patients also reported higher pain scores. Bilateral surgeries showed slightly higher pain levels, though not statistically significant. Postoperative complications were rare (one case of paraesthesia, two infections).

**Conclusions:**

Postoperative pain after third molar surgery performed by GDPs was highest on the day of surgery and declined rapidly.

** Key words:**Postoperative pain, third molar, oral surgery.

## Introduction

Tooth impaction is when a tooth fails to attain a normal functional position in the oral cavity. Thirds molars are quite prone to impaction. The worldwide prevalence of third molars impaction is estimated to be 24%, with mandibular teeth having 57.6% higher odds than maxilla teeth [1].

Individuals with impacted third molars are prone to conditions like pericoronitis, periodontal lesions of adjacent molar, root resorptions and dental caries [2]. Cysts and tumours are less prevalent [3]. Surgical removal of impacted third molars is a common treatment approach with typical transient postoperative complications like pain, bleeding, swelling and infection of the operation site [4].

Postoperative pain is by experience the most dreaded postoperative morbidity by patients and a known cause of preoperative anxiety. Several randomized controlled studies have been conducted evaluating the effect of different operative techniques on postoperative pain in hopes of minimizing postoperative morbidity [5]. Evidence-based information on postoperative pain intensity and pattern conveyed to patients may ease preoperative anxiety and facilitate informed consent [6].

Previous studies on postoperative pain following third molar surgery have predominantly been conducted in specialist clinics by oral and maxillofacial surgeons. However, there is a notable lack of cohort studies focusing on pain outcomes in procedures performed by general dental practitioners (GDPs). This study aimed to address this gap by evaluating postoperative pain intensity over the first seven days following the surgical removal of impacted mandibular third molars by GDPs.

## Material and Methods

A prospective cohort study was carried out in three public dental care clinics of Region Norrbotten (Norrbotten’s county council) in Luleå, Sweden, between October 2022 to December 2023. Individuals were included if they were ≥18 years old with a planned surgical removal of at least one impacted mandibular third molar. Individuals were excluded if they: were incapable of giving informed consent; had already participated in the study; had an American Society of Anesthesiologists physical status classification ≥ 3; were pregnant; had an extraction of the maxillary molar planned on the same side and day as the mandibular third molar surgery; had acute pain in the third mandibular molar; ought to be treated by an oral maxillofacial surgeon. The reasons for referral to an oral maxillofacial surgeon were: radiological signs indicating a risk of injury to inferior alveolar nerve (IAN); head and neck irradiated; treated with antiresorptive or antiangiogenic medications associated with an increased risk for osteonecrosis of the jaw (e.g. bisphosphonates); severe neutropenic patients (<0.5x109/L), and individuals with defective granulocyte function; or high risk of haemorrhage due to thrombocytopenia <30x109/L, liver disease or other diagnosed bleeding disorders.

- Procedure

All mandibular third molar surgeries were performed by four GDPs with good but different levels of clinical experience (5, 8, 15 and 35+ years respectively). Individuals were given 1g of paracetamol pre-operative. Peroral conscious sedation with midazolam 1mg/ml was available for individuals with anxiety. Local anaesthesia was administered using 2% Xylocaine adrenaline 12.5µg/ml, or combining with 3% Citanest Octapressin 0.54µg/ml or 4% Septocaine adrenaline 5µg/ml. The mouth was rinsed for one minute with 0.12% chlorhexidine digluconate solution. Sterile field was set up. The GDP made an incision gingivally with a relief incision towards the anterior border of the mandible ramus and elevated the full thickness mucoperiosteal flap. Osteotomy was performed when necessary, with a conventional rotatory presterilized round bur connected to a dental drill unit (Nobel Biocare OsseoSetTM 200 or KaVo INTRAsurg® 300) up to 40,000rpm with copious irrigation with sterile saline solution. If required, the tooth was sectioned with surgical burs. After tooth removal, the GDP removed any granulation and/or follicular tissue with surgical curettes and evened out bone irregularities with surgical round burs. The socket was thoroughly irrigated with sterile saline solution and inspected before the flap was re-approximated and sutured with synthetic multifilament resorbable Vicryl 3-0 or 4-0 sutures.

Patients received postoperative information regarding management of postoperative complications. GDPs prescribed 1g of paracetamol three to four times per day combined with 500mg naproxen twice a day. Individuals who took other medications which could interact with naproxen were prescribed etoricoxib 90 mg (selective COX-2 inhibitor) one per day for three days. Individuals with non-steroidal anti-inflammatory drug (NSAID) allergies or contraindications were only prescribed paracetamol. All participants could request stronger analgesics during the seven days observation period. No antibiotics were administered prior to or after surgery. Patients were advised to use 0.12% Chlorhexidine mouth rinse twice a day.

The pain was reported using a numerical rating scale (NRS) from 0-10 were 0 stood for “no pain”, 1 – 3 “mild pain”, 4 – 6 “moderate pain”, 7 – 9 “severe pain”, and 10 for “worst pain imaginable” [7]. Participants were instructed to document their post-operative pain experience for seven days, every 24 hours, from the end of the operation in a questionnaire. The first pain score registration was their worst pain felt on the day of surgery (Day 0). Participants were advised to set phone alarms as a reminder to fill in their questionnaires every 24 hours and YAK also sent reminders to participants. Participants were also instructed to document the number and kind of painkillers taken, and to use of Chlorhexidine as mouthwash.

A follow-up visit was scheduled on the 8th day after surgery to examine the surgical wound healing process. Participants submitted their questionnaires during their follow up visit, or posted, or used an online variant. If patients gave incorrect and inadequate answers or did not respond, they were contacted to clarify possible irregularities.

During the observation period, participants who self-medicated with antibiotics were excluded as well as those with signs of intra-operative nerve injury.

- Variables of interest

The following variables were recorded by the GDPs: sex, age, tobacco use, oral hygiene status categorized as good, acceptable or bad based on visibility of dental plaque (no visible plaque, visible dental plaque less than 50% and visible plaque more than 50% of teeth surfaces), indication of the operation, tooth angulation, degree of surgical difficulty to remove mandibular third molars according to Pedersons index [8] need of conscious sedation with Midazolam, type of operation (whether unilateral or bilateral), operation technique (simple luxation, or whether osteotomy, and/ or tooth separation were needed), intra-operative complications and operation working time (i.e. time from first incision to last suture).

- Statistics

A formal calculation of the minimum sample size was not feasible due to limited information regarding the characteristics of the primary outcome. However, a comparable study by Seymour *et al*. included 80 participants [9]. Taking practical factors into account, a minimum sample size of 100 participants was deemed adequate for statistical analysis.

Data were analysed using descriptive statistics, chi-square tests, and t-tests. IBM SPSS statistics version 29.0 was used for these analyses.

## Results

Of the 162 individuals screened, 152 were eligible (i.e., they met the inclusion criteria and did not meet any exclusion criteria), and of those, 133 agreed to participate. Patients that submitted questionnaires were 111. However, three questionnaires were not analysed because participants self-medicated with antibiotics (*n*=2), or paraesthesia occurred in the nervus alveolaris inferior innervation area (*n*=1).

Descriptive statistics and demographic information are presented in  Table 1. Participants ranged in age from 18 to 72 years, with a mean age of 24.09 years (standard deviation [SD] = 7.06).

Pain intensity over time, summarized in  Table 2, peaked on Day 0, when 4% reported the worst pain imaginable (score 10), 34% reported severe pain [7-9], and 35% reported moderate pain [4-6]. By Day 1, pain dropped significantly to mild levels (*p* < 0.001) and continued to decrease throughout the week. By Day 6 and 7, 25.7% and 31.4% of participants, respectively, reported no pain.

 Table 3 presents mean pain scores by sex, sedation, and operation type—factors more commonly observed with NRS 10 scores. Females reported significantly higher pain on Days 2-4 and used more analgesics. Sedated patients also reported higher pain. Bilateral surgeries showed slightly higher pain scores, though not statistically significant. No other variables correlated clearly with pain. Postoperative complications (paraesthesia, *n*=1; infection, *n*=2) were excluded from analysis.

Patients consumed the highest amount of analgesic during the first 24 hours after surgery. Consumption decreased successively for the rest of the week. Female participants consumed more analgesics than male participants both paracetamol and naproxen on all days, also statistically significant for days 2, 3 and 4 with *p*<0.05. The reported mean pain scores and consumption of analgesics for bilateral operations were slightly higher than for unilateral operations for all days (except day 3 for mean pain), but no statistical significance was found.

## Discussion

The study aligns with previous research, showing that postoperative pain after third molar surgery is typically short-lived, peaking early and gradually subsiding, along with a decreasing need for analgesics. The noticeable decrease in pain one day after surgery may be due to lower nociception, elevated cortisol levels, and effective analgesia [10]. These findings are consistent with Seymour *et al*., despite differences in setting and operator background [9]. While their procedures were performed by specialists in a university hospital, this study involved GDPs—highlighting that comparable outcomes can be achieved, likely reflecting the practitioners’ surgical proficiency and adherence to hygiene protocols, despite individual variation. The results suggest that a substantial number of patients undergo third molar surgery under the care of GDPs.

Females reported higher pain scores for all seven days, which concurs with the results of Seymour *et al*., who interpreted that females have greater pain sensitivity [9]. They also suggested that females might have been better at recording their pain, while males might have under-reported their pain scores. Seymour *et al*. did not consider the consumption of analgesics, whereas this study reported that female participants consumed more analgesics for all seven days, implying a higher pain experience for females [9]. This is also in accordance with previous studies that suggested sex as a predictive factor for postoperative pain [11].

Sedated patients reported higher pain scores, though the difference was statistically significant for only one day. This study does not provide sufficient evidence of a direct association between sedation and increased postoperative pain; any observed link is more likely explained by selection bias. Previous research has found that higher levels of preoperative anxiety are associated with increased postoperative pain [12]. In our study, clinical records showed that the sedated patients were anxious prior to the procedure, which may help explain the need of sedation. Moseley *et al*. suggest that understanding pain can reduce fear and anxiety, potentially mitigating the pain experience [13].

Although bilaterally operated patients reported slightly higher pain scores, as expected, these differences were not statistically significant. While previous studies have suggested that bilateral third molar surgery may be associated with an increased risk of postoperative infections and nerve injury, our findings indicate that such complications were relatively uncommon [14]. Of the 133 participants, three required antibiotics for postoperative infections—two after bilateral procedures—and one case of paraesthesia followed the removal of both mandibular third molars in a single session. These infrequent complications support the safety of surgeries performed by general dental practitioners (GDPs), likely due to appropriate case selection and referrals. However, larger studies are needed to better assess the risks of bilateral procedures.

A strength of the study is the exceptionally high participation rate (87.5%) and the response rate for pain questionnaires among participants (83.5%). The relatively large sample size and inclusion of multiple clinicians with varying experience across different clinics enhanced external validity. Efforts were made to minimize bias, though objectively assessing compliance in participants with neuropsychiatric diagnoses proved challenging. The use of the NRS, a well-validated and reliable unidimensional pain scale is another strength [7].

A key limitation was the potential for missing data, which could impact internal validity; this was mitigated by over-recruiting and implementing strategies to improve compliance. However, the need for these measures remained minimal. Additional limitations included an all-female clinician group, and a mid-study change in anaesthetics, though the latter did not impact outcomes.

The findings of this study contribute to improving patient understanding of acute postoperative pain patterns, supporting informed consent, reducing preoperative anxiety, and promoting better recovery within a GDP setting. They also offer clinicians valuable insights for optimizing treatment planning and pain management, while laying the groundwork for future intervention studies.

## Conclusions

Postoperative pain after mandibular third molar surgery by GDPs peaks within 24h after surgery and declines significantly afterwards. Some weak associations of severe pain and some variables (sex, sedation) were found but they should be judged cautiously.

## Figures and Tables

**Table 1:** Descriptive statistics and demographics of the N=133 patients in the study recorded by GDPs.

Variables	Categories	Number of cases, (%)
Sex	Male Female	75 (56) 58 (44)
Tobacco use	No Snuff Cigarettes Snuff and cigarettes	96 (72) 27 (20) 5 (4) 5 (4)
Oral hygiene	Good (no visible plaque in teeth surfaces) Acceptable (plaque < 50% surfaces) Bad (plaque >50% surfaces)	112 (84) 15 (11) 6 (5)
Indication for operation	Pericoronitis Dental caries Periodontitis Resorption Prophylactic	51 (39) 12 (9) 56 (42) 3 (2) 12 (9)
Tooth angulation	Vertical Mesioangular Horizontal Distoangular	26 (20) 46 (35) 50 (38) 11 (8)
Degree of difficulty (Pederson's index) (8)	Easy Moderate Difficult	38 (29) 80 (60) 15 (11)
Conscious sedation	Yes No	44 (33) 89 (67)
Type of operation	Unilateral Bilateral	98 (74) 35 (26)
Operation technique	Simple luxation Osteotomy Tooth separation	16 (12) 128 (96) 117 (88)
Intra-operative complications	None Soft tissue damage Root fracture Severe haemorrhage	125 (95) 1 (1) 4 (3) 2 (2)
Operation time (minutes)	< 30 30 - 45 46 - 60	104 (78) 23 (17) 6 (5)

† - Multiple responses

**Table 2:** Summary measure of participants reported pain scores based on NRS 0-10. SD- Standard deviation.

	Day 0	Day 1	Day 2	Day 3	Day 4	Day 5	Day 6	Day 7
Valid (n)	101	106	105	104	103	102	101	102
Proportion with pain score 7-10 (%)	38	11	9	12	7	6	5	2
Mean	5.20	3.11	2.96	3.05	2.68	2.29	2.19	1.72
Median	5	3	3	3	2	2	2	1
SD	2.64	2.31	2.27	2.42	2.25	2.17	1.95	1.70
Range	0-10	0-10	0-8	0-9	0-8	0-10	0-8	0-8

SD- Standard deviation

**Table 3:** Mean pain scores, standard deviation (SD) for males and females, bilateral and unilateral operation and conscious sedation on days 0 -7 post-surgery. P-value of Two-sided T-test for equality of means. 95% Confidence interval and Significance value 0.05. Comparison of means for each category was computed taking into consideration missing data. Total individuals with at least one score n=108.

		Day 0	Day 1	Day 2	Day 3	Day 4	Day 5	Day 6	Day 7
Sex	Male (SD)	4.85 (2.63)	2.92 (2.20)	2.36 (1.99)	2.45 (2.18)	2.12 (1.93)	1.96 (2.05)	2.07 (2.03)	1.63 (1.70)
	Female (SD)	5.60 (2.63)	3.36 (2.44)	3.70 (2.39)	3.80 (2.51)	3.37 (2.42)	2.71 (2.26)	2.34 (1.84)	1.83 (1.71)
	P-value	0.159	0.326	0.002*	0.004*	0.005*	0.085	0.491	0.555
Bilateral operation	Bilateral (SD)	5.97 (2.97)	3.41 (2.74)	3.14 (2.20)	2.93 (1.94)	2.71 (2.09)	2.50 (2.43)	2.41 (2.01)	1.93 (1.98)
	Unilateral (SD)	4.97 (2.49)	3.00 (2.14)	2.89 (2.30)	3.09 (1.58)	2.67 (2.31)	2.22 (2.08)	2.11 (1.93)	1.64 (1.59)
	P-value	0.167	0.415	0.626	0.761	0.924	0.558	0.498	0.440
Conscious sedation	Yes (SD)	5.65 (2.74)	3.44 (2.40)	3.44 (2.20)	3.79 (2.57)	3.09 (2.20)	2.82 (2.35)	2.59 (2.06)	2.12 (1.99)
	No (SD)	4.97 (2.58)	2.96 (2.27)	2.73 (2.28)	2.69 (2.27)	2.48 (2.25)	2.04 (2.05)	1.99 (1.87)	1.51 (1.52)
	P-value	0.225	0.318	0.135	0.029*	0.196	0.092	0.142	0.092

## Data Availability

The datasets used and/or analyzed during the current study are available from the corresponding author.
